# Do the Most Attractive Faces of Patients With Class II Division 1 Malocclusion Differ From Those With the Least Attractive Faces in Terms of Angular and Proportional Measurements Assessed on Frontal and Lateral Photographs?

**DOI:** 10.7759/cureus.33455

**Published:** 2023-01-06

**Authors:** Amineh Adi Mortada, Ahmad S Burhan, Mohammad Y Hajeer, Fehmieh R Nawaya, Ghaith F Sahtout

**Affiliations:** 1 Department of Orthodontics, University of Damascus Faculty of Dentistry, Damascus, SYR; 2 Department of Pediatric Dentistry, Syrian Private University Faculty of Dentistry, Damascus, SYR

**Keywords:** attractiveness, panel of assessors, skeletal class ii division 1 malocclusion, frontal photographs, lateral photographs, facial proportions, facial esthetics, photographs, class ii malocclusion, facial measurements

## Abstract

Background

This study investigated the facial angles and proportions affecting facial aesthetics in patients with skeletal class II division 1 malocclusion between those labeled the most attractive and least attractive in each gender.

Methodology

The study sample included pretreatment extraoral photographs of 60 patients (30 males and 30 females) with skeletal class II division 1 malocclusion according to the ANB angle aged between 18 and 21 years. A panel of 240 laypersons (aged 20-25 years; the average age of 22.5 ± 0.37 years; 120 males and 120 females) scored the aesthetic evaluation of photographs using the visual analog scale (VAS). Two groups were created according to the mean aesthetic scores of each photograph, namely, the most attractive group with the highest aesthetic scores, and the least attractive group with the least aesthetic scores. A total of 12 patients in each group were selected. Subsequently, their angular and proportional measurements on the frontal and lateral photographs were calculated. Independent-sample t-tests were used to determine if there were significant differences in these measurements between the two groups.

Results

There was no significant difference in frontal variables between the most attractive and least attractive groups in each gender. The angle NPog-FH was significantly greater in the most attractive males than in the least attractive males, while there was no significant difference between the most attractive and least attractive females regarding any of the profile variables.

Conclusions

The most attractive females with class II division 1 were similar to the least attractive on evaluating the frontal and profile variables. In contrast, the most attractive males with class II division 1 malocclusion had more protrusion in the chin than the least attractive male patients, with no differences in other profile and frontal variables. These findings suggest considering the chin position during the diagnosis and treatment planning of class II division 1 malocclusion patients.

## Introduction

Facial attractiveness is defined as the appearance and proportions of the face that contribute positively to a female’s femininity and a male’s masculinity [1]. It is considered an essential factor in social acceptance and popularity [2,3].

Facial attractiveness is evaluated subconsciously and affects everyday interactions such as individual socioeconomic status, job position opportunities [4], and robust interpersonal connections [5]. Unattractive people are perceived to have lower social competency and are less friendly [6]. On the other hand, unattractiveness decreases self-esteem and a person’s feelings of self-image [7].

Aesthetic improvement is the main reason adults seek orthodontic treatment [8]. Thus, achieving patients’ desired esthetic appearance with a proper dental-skeletal relation results in acquiring social and psychological benefits for the patients. Therefore, improving the facial attractiveness of the patients is the primary goal of orthodontic treatment [9]. However, public preferences do not necessarily concur with orthodontic standards [10]. Thus, the professional assessment of treatment outcomes may not be related to patient satisfaction [11].

Soft-tissue esthetic assessment is essential in diagnosing and formulating orthodontic treatment plans [12]. Orthodontists should know the characteristics that positively or negatively affect facial appearance and consider them in treatment planning. Failure to address the sources of the negative facial appearance would result in the aesthetic failure of the treatment plan. Therefore, when planning orthodontic treatment, the aesthetic perception of the patients and their societies should agree with the orthodontic standards [13]. Further, the patient should participate in the decision-making and treatment outcome assessment [14].

Photogrammetry is highly recommended for large epidemiological studies due to equipment availability, low cost, and time-saving [15]. Moreover, photographs are considered the closest representation of the daily perception of facial features [16]. The view angle of the face affects the perception of attractiveness [17]. Therefore, orthodontists commonly use the records of frontal, frontal smiling, and lateral photographs for diagnosis, facial esthetic analysis, and treatment planning [18]. Stereophotogrammetry has also been used successfully in assessing patients with dentofacial deformities, but its use is limited due to the cost of the device [19,20].

Dentofacial deformities have been reported as a reason for decreased social attractiveness [21]. Akan et al. evaluated the facial attractiveness using frontal photographs for 335 untreated Turkish adolescents categorized according to ANB angle into class I (174), class II (125), and class III (36). They concluded that 43% of the most attractive patients had skeletal class I, 43% had skeletal class II, and 14% had skeletal class III deformities. They indicated that sagittal skeletal discrepancies did not affect frontal facial attractiveness [22]. Macías et al. studied the perception of facial aesthetics in a young Spanish population. The evaluation was done using frontal relaxing, frontal during a smile, and profile photographs. They noted that individuals with skeletal class 1 malocclusion are more attractive than those with skeletal class II and III malocclusion. Moreover, they concluded that faces with severe and moderate class II or class III malocclusion were considered unattractive [23].

Class II malocclusion is a prevalent sagittal jaw discrepancy and constitutes many patients who refer to orthodontic treatment [24,25]. Moderate class II malocclusion treatment in adults is usually done by camouflage. Achieving aesthetic results in these cases is difficult because the camouflage corrects the dental occlusion with little influence on facial characteristics [22]. Many previous studies have shown that some orthodontic treatments do not necessarily change aesthetic outcomes [16,26-28], and having ideal measurements is not essential for facial attractiveness [29,30]. Thus, for the best aesthetic results, it is fundamental to know the unpleasant facial characteristics that should be improved with orthodontic treatment and the nice features that should be achieved. Therefore, quantitatively identifying these characteristics is essential for clinical application. Therefore, this study aimed to investigate the facial measurements that differ between the most and least attractive patients with skeletal class II division 1 malocclusion in each gender.

## Materials and methods

Study design and settings

This was a cross-sectional study for descriptive and analytical purposes, and the collected data were based on patients’ photographs. This study was conducted at the Department of Orthodontics, the Faculty of Dentistry, University of Damascus (Damascus, Syria) between April 2019 and October 2021. Ethical approval was obtained from the Local Research Ethics Committee at the Faculty of Dentistry, University of Damascus (reference number: UDDS-999-27032019/SRC-1450). This project was funded by the University of Damascus Postgraduate Research Budget (MMY08945209DEN).

Sample size calculation

The sample size was calculated using Minitab® version 17 (Minitab Inc., State College, Pennsylvania, USA). One of the main variables used in assessing facial profile was the facial convexity angle. A difference of 5 degrees between the two groups under evaluation (i.e., the most attractive persons versus the least attractive persons) was considered clinically significant. The standard deviation of this variable from a previous study was 3.88 degrees [31]. Employing a two-sample t-test with a power of 0.85 and an alpha level of 0.05, the required sample size was 12 persons in each group (i.e., a total of 24 patients). However, to differentiate between those who were expected to have high scores in terms of attractiveness from those who were expected to have the lowest scores (i.e., the least attractive persons), it was postulated that some persons should be placed in the middle between the two groups. Therefore, we added six patients to the 24 required ones to achieve a sample size of 30 persons. According to the goals of this research, 60 patients were required to constitute two groups based on gender (i.e., 30 female patients and 30 male patients).

Sample collection and patient recruitment

A total of 127 class II patients who attended the Orthodontic Department at the Faculty of Dentistry, University of Damascus were examined. In total, 86 patients met the inclusion criteria. The aim and methodology of the study were explained to each patient using an information sheet, and informed consent was obtained in the case of acceptance. A total of 86 patients were accepted to participate, and 60 were randomly selected from the sample using a computer-generated sampling method to ensure equal distribution for males and females (i.e., 30 males and 30 females).

The following Inclusion criteria were employed: age between 18 and 21 years, skeletal class II division 1 malocclusion resulting from mandibular retrognathia (SNB angle = 73°-75°), the sagittal skeletal discrepancy angle (ANB angle) between 5° and 7°, overjet between 5 mm and 7 mm, close-to-normal growth pattern (Bjork sum = 396 ± 6), the presence of all permanent teeth (excluding third molars), and no previous orthodontic treatment. Exclusion criteria were severe skeletal class II cases (ANB >7°), clear vertical growth pattern with Bjork sum >402 or horizontal growth pattern with Bjork sum <390, dental or facial bruises, surgical or cosmetic work on the maxillofacial area, and presence of a craniofacial syndrome.

Photographing method

The 60 patients were photographed in three positions, namely, frontal relaxed, frontal during a smile, and relaxed profile using a Nikon D80 camera (Nikon D80; Nikon Corporation, Tokyo, Japan), 10.2 megapixels, and a 70-200 mm macro lens. The patient stood straight with arms freely positioned on both sides of the body [32], taking into consideration the normal position of the head, as described by Moorrees et al. [33]. A mirror with a movable base was placed opposite the patient to achieve this position. Patients were asked to fix their gaze on the horizontal line drawn on the mirror, which represented the line passing between the pupils of the eyes and parallel to the ground with their lips resting. Photographs were taken with a white background to avoid the influence of the surrounding colors on the aesthetic evaluation of the photographs. The camera was mounted on a camera mount at a distance of 150 cm from the N point in the frontal photographs and the Po point in the profile ones. The photographs were converted to black and white using the Photos program version 2018.18011.15918.0 (Photos, Microsoft Corp., Seattle, WA, USA). All photographs were taken by one researcher (AAM).

The evaluation panel

An evaluation panel of 240 laypersons was selected randomly from the first three years of students from the Faculty of Medicine at the University of Damascus. Their age ranged between 18 and 22 years from both genders equally, they had no connection with the patients, and they were unfamiliar with orthodontics and aesthetics. The 240 laypersons rated the 60 patient photograph sets.

The evaluation procedures of patients’ photos

The photograph sets of each patient were randomly placed into Microsoft PowerPoint 2016 slide show program (Microsoft Corp., Seattle, WA, USA), and their size was fixed at 13.2 × 8.5 cm. Each slide, including a patient set, was displayed for 20 seconds on a laptop (HP, California, USA), with an interval of 10 seconds after every 15th slide. The panel members were instructed to rate the facial appearance regardless of the photo color using the visual analog scale (VAS) from 0 to 100 mm on the rating sheet (0 being the least attractive, and 100 being the most attractive), keeping in mind that the score of 50 was the average degree of facial aesthetics.

The mean evaluation score for each patient photograph set was calculated, and the 60 patients were ranked according to their means. The 12 most attractive and 12 least attractive photographs for each gender were selected.

The photographic analysis of facial soft tissues

**Figure 1:**
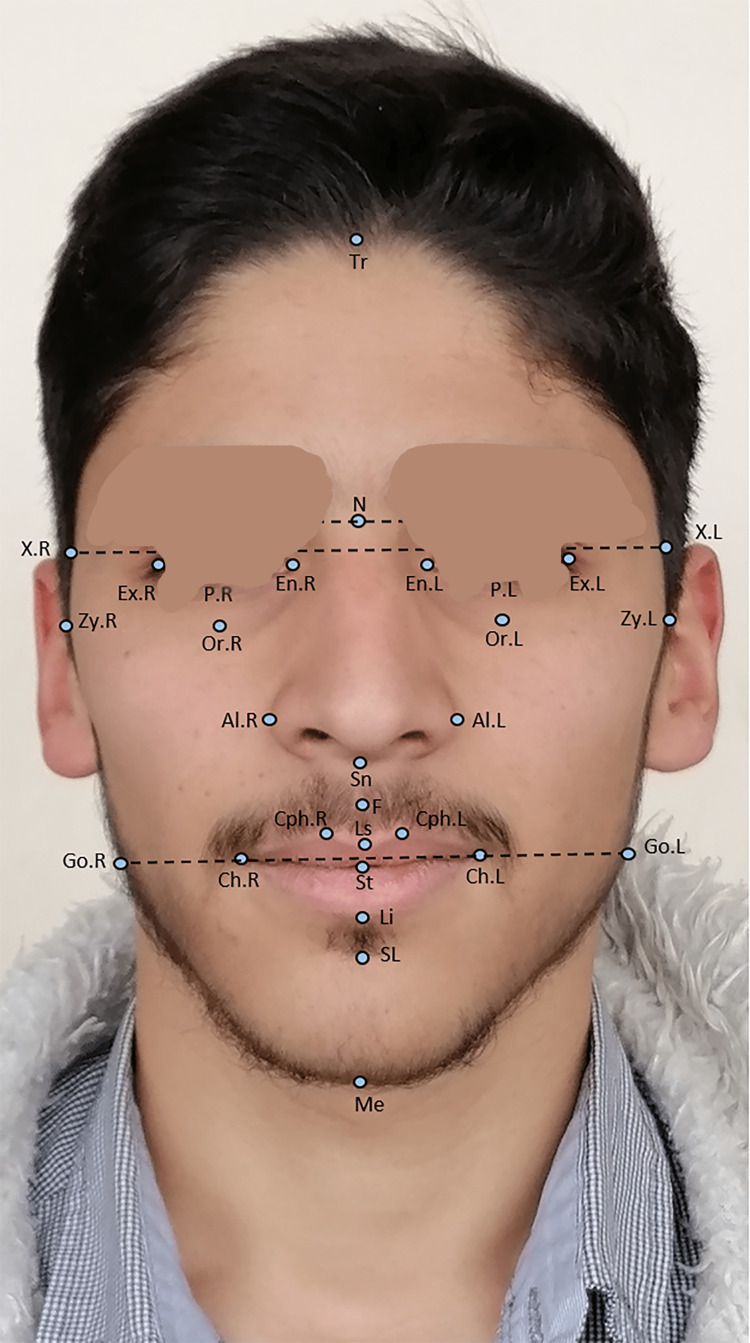
Landmarks used on the frontal photograph. Tr = trichion point at the hairline; N = nasion point in the midline at the level of the line connecting the highest points of the superior palpebral sulci; En.R = endocanthion on the right side; En.L = endocanthion on the left side; Ex.R = exocanthion on the right side; Ex.L = exocanthion on the left side; P.R = middle of the pupil on the right side; P.L = middle of the pupil on the left side; Or.R = infraorbitale point on the right side; Or.L = infraorbitale point on the left side; Al.R = the most lateral point of the nose on the right side; Al.L = the most lateral point of the nose on the left side; Sn = subnasale point at the base of the columella and upper lip junction; F = philtrum point at the superior labial sulcus; Ls = labrale superior; Li = labrale inferior; St = stomion; Ch.R = cheilion point on the right side; Ch.L = cheilion point on the left side; Cph.R-L = crista-philtrum points at the top of vermilion edge of the upper lip; SL = sublabial point at the inferior labial sulcus; Me = menton point; X.R and X.L = constructed points at bipupil line; Zy.R = zygion on the right side; Zy.L = zygion on the left side; Go.R and Go.L = constructed points at stomion

**Figure 2:**
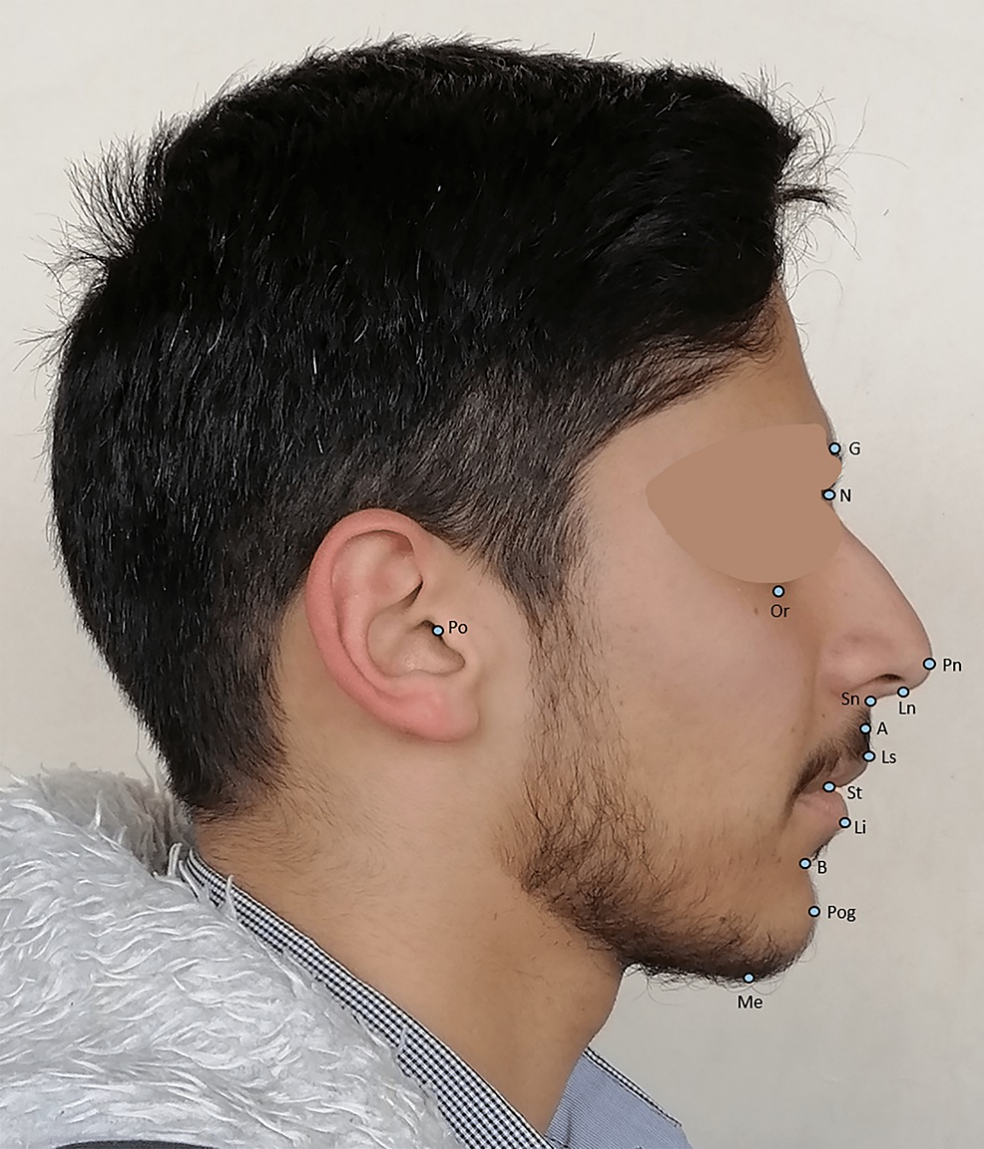
Landmarks used on the lateral photograph. G = Glabella point at the upper edge of the eyebrows; N = nasion point; Pn = pronasale point; Ln = lowest nose point; Sn = subnasale point at the base of the columella and the upper lip junction; A = the deepest point at the superior labial sulcus; Ls = labrale superior; St = stomion; Li = labrale inferior; B = the deepest point at the inferior labial sulcus; Pog = Pogonion point; Me = menton point; Or = infraorbitale point; Po = porion point at the uppermost point on the tragus

The frontal and lateral photographs taken of the face at a rest position of the selected patients were evaluated; 19 points on the frontal photograph, with definitions given elsewhere [29,34] (Figure 1), and 14 points on the lateral photograph. The definition of the landmarks used in the lateral photograph is given by Kiekens et al. [29] (Figure 2). Twenty-four ratios and four angles on frontal photos were calculated. The definition of these ratios and angles is based on previous studies [35,36], and two ratios and 11 angles on lateral photographs were measured manually. The definitions of measurements made on the lateral photographs are given by two previous studies [37,38]. All of these measurements were judged essential in quantitating the facial soft-tissue esthetics. The definitions of the variables analyzed in the frontal photographs are given in Table 1, whereas those analyzed in the lateral photographs are given in Table 2.

**Table 1:** Definitions of the variables used in the analysis of frontal photographs. Variable definitions are taken from Farkas et al. [35] and Koury and Epker [36].

Variable	Definition
Tr-N/N-Me	Forehead height/middle and lower facial height
N-Sn/Tr-Me	Nasal height/total facial height
N-Sn/N-Me	Nasal height/middle and lower facial height
AlR-AlL/N-Sn	Nasal width/nasal height
AlR-AlL/ChR-ChL	Nasal width/mouth width
AlR-AlL/XR-XL	Nasal width/facial width
EnR-EnL/XR-XL	Inter-endocanthal width/facial width
EnR-EnL/ExR-ExL	Inter-endocanthal width/inter-exocanthal width
EnR-EnL/AlR-AlL	Inter-endocanthal width/nasal width
ChR-ChL/ExR-ExL	Mouth width/inter-exocanthal width
Ls-St/St-Li	Upper vermilion height/lower vermilion height
Ls-St/Sn-St	Upper vermilion height/upper lip height
St-Li/St-SL	Lower vermilion height/lower lip height
St-SL/Sn-St	Lower lip height/upper lip height
Sn-St/ChR-ChL	Upper lip height/mouth width
Cph-Li/ChR-ChL	Upper and lower vermilion height/mouth width
Sn-St/Sn-Me	Upper lip height/lower facial height
Sn-Me/N-Me	Lower facial height/middle and lower facial height
Sn-Me/Tr-Me	Lower facial height/total facial height
ChR-ChL/Sn-Me	Mouth width/lower facial height
XR-XL/Tr-Me	Facial width/total facial height
N-Me/XR-XL	Middle and lower facial height/facial width
Sn-Me/XR-XL	Lower facial height/facial width
ChR-ChL/XR-XL	Mouth width/facial width
ZyR-Me-ZyL	Upper facial width angle
GoR.Me.GoL	Mandibular width angle
ExR-Me-ExL	Facial aperture modified angle
N.F-Sn.Me	Facial symmetry angle (angle between the facial midline and subnasal menton line)

**Table 2:** Definitions of the variables used in the analysis of lateral photographs. Variable definitions are taken from Holdaway et al. [37] and Nguyen [38].

Pn-N-Pog	The angle between pronasale and pogonion
a1	The angle between the nostril axis and the horizontal plane
Pn-N/N-Sn	The nasal tip prominence (horizontal distance between pronasale and perpendicular from point nasion)/nasal height
Sn-Pn/Sn.St	The horizontal distance between subnasale and pronasale/upper lip height
Cm-Sn-Ls	Nasolabial angle
a2	The angle between the horizontal plane and tangential line to labrale superius
Li-B-Pog	Labiomental angle
Sn-Ls.B-Li	Interlabial angle (upper and lower lip protrusion)
Po-N-Pog	Mandibular protrusion (angle between the pogonion and porion nasion line)
B-Pog.FH.p	Chin protrusion (Angle between B-pogonion and a line parallel to the FH line)
A-N-B	Soft-tissue ANB
G-Sn-Pog	Facial convexity angle
N-Pog-FH	The angle between the facial plane and the Frankfort plane

Error of methods

The variables were again measured on the frontal and lateral photographs of 24 randomly selected patients after one month by the same researcher. The first and second readings were compared using the paired-sample t-test. The intraclass correlation coefficients (ICCs) were applied to evaluate the intraobserver reliability.

Statistical analysis

SPSS version 20 (IBM Corp., Armonk, NY, USA) program was used to perform all statistical analyses. The normality of the data distribution was determined using the Shapiro-Wilk test. An independent-sample t-test was applied to evaluate the differences between the most and the least attractive groups. The significance level was set at 0.05 and then adjusted using Bonferroni correction for multiple comparisons. Therefore, the significance level was 0.001 in comparing frontal variables (28 comparisons) and 0.003 in profile variables (13 comparisons). For each variable, the percentage difference of the values between the most and least attractive groups was obtained according to the following equation: (μ1-μ2)/μ2 × 100, where μ1 represents the mean value of the variable in the most attractive group, and μ2 represents the mean value of the variable in the least attractive group.

## Results

The error of the method and reliability of the measurements

The error of the method using the paired-sample t-test showed no statistically significant differences in the measurements between the first and second times for all frontal and lateral measurements (p > 0.05). The ICCs showed that the frontal ratios (AlR-AlL/ChR-ChL and cph-Li/ChR-ChL) and the profile angle (Cm-Sn-Ls) had the lowest ICC values (0.812). In contrast, the frontal ratio (EnR-EnL/XR-XL) and the profile angle (Li-B-Pog) had the highest values (0.993) (Tables 3, 4).

**Table 3:** The error of method for frontal variables using the intraclass correlation coefficients (ICCs) and paired-sample t-test. *: P-value for ICC, †: P-value for paired-sample t-test. Tr-N/N-Me = forehead height/middle and lower facial height; N-Sn/Tr-Me = nasal height/total facial height; N-Sn/N-Me = nasal height/middle and lower facial height; AlR-AlL/N-Sn = nasal width/nasal height; AlR-AlL/ChR-ChL = nasal width/mouth width; AlR-AlL/XR-XL = nasal width/facial width; EnR-EnL/XR-XL = inter-endocanthal width/facial width; EnR-EnL/ExR-ExL = inter-endocanthal width/inter-exocanthal width; EnR-EnL/AlR-AlL = inter-endocanthal width/nasal width; ChR-ChL/ExR-ExL = mouth width/inter-exocanthal width; Ls-St/St-Li = upper vermilion height/lower vermilion height; Ls-St/Sn-St = upper vermilion height/upper lip height; St-Li/St-SL = lower vermilion height/lower lip height; St-SL/Sn-St = lower lip height/upper lip height; Sn-St/ChR-ChL = upper lip height/mouth width; Cph-Li/ChR-ChL = upper and lower vermilion height/mouth width; Sn-St/Sn-Me = upper lip height/lower facial height; Sn-Me/N-Me = lower facial height/middle and lower facial height; Sn-Me/Tr-Me = lower facial height/total facial height; ChR-ChL/Sn-Me = mouth width/lower facial height; XR-XL/Tr-Me = facial width/total facial height; N-Me/XR-XL = middle and lower facial height/facial width; Sn-Me/XR-XL = lower facial height/facial width; ChR-ChL/XR-XL = mouth width/facial width; ZyR-Me-ZyL = upper facial width angle; GoR.Me.GoL = mandibular width angle; ExR-Me-ExL = facial aperture modified angle; N.F-Sn.Me = facial symmetry angle (angle between the facial midline and the subnasal-menton line)

Variables	N	ICC	P-value^*^	P-value^†^
Tr-N/N-Me	24	0.956	<0.001	0.886
N-Sn/Tr-Me	24	0.916	<0.001	0.824
N-Sn/N-Me	24	0.986	<0.001	0.636
AlR-AlL/N-Sn	24	0.863	<0.001	0.615
AlR-AlL/ChR-ChL	24	0.812	<0.001	0.673
AlR-AlL/XR-XL	24	0.976	<0.001	0.744
EnR-EnL/XR-XL	24	0.993	<0.001	0.991
EnR-EnL/ExR-ExL	24	0.837	<0.001	0.645
EnR-EnL/AlR-AlL	24	0.954	<0.001	0.929
ChR-ChL/ExR-ExL	24	0.943	<0.001	0.961
Ls-St/St-Li	24	0.834	<0.001	0.794
Ls-St/Sn-St	24	0.925	<0.001	0.677
St-Li/St-SL	24	0.972	<0.001	0.844
St-SL/Sn-St	24	0.922	<0.001	0.64
Sn-St/ChR-ChL	24	0.973	<0.001	0.853
Cph-Li/ChR-ChL	24	0.812	<0.001	0.936
Sn-St/Sn-Me	24	0.927	<0.001	0.686
Sn-Me/N-Me	24	0.986	<0.001	0.953
Sn-Me/Tr-Me	24	0.926	<0.001	0.662
ChR-ChL/Sn-Me	24	0.844	<0.001	0.911
XR-XL/Tr-Me	24	0.874	<0.001	0.967
N-Me/XR-XL	24	0.944	<0.001	0.743
Sn-Me/XR-XL	24	0.986	<0.001	0.929
ChR-ChL/XR-XL	24	0.825	<0.001	0.792
ZyR-Me-ZyL	24	0.836	<0.001	0.742
GoR.Me.GoL	24	0.975	<0.001	0.831
ExR-Me-ExL	24	0.825	<0.001	0.857
N.F-Sn.Me	24	0.823	<0.001	0.896

**Table 4:** The error of method for profile variables using the intraclass correlation coefficients (ICCs) and paired-sample t-tests. *: P-value for ICC, †: P-value for paired-sample t-test. Pn-N-Pog = the angle between pronasal and pogonion; a1 = the angle between the nostril axis and horizontal plane; Pn-N/N-Sn = nasal tip prominence ratio (horizontal distance between pronasal and perpendicular from point nasion)/nasal height; Sn-Pn/Sn.St = the horizontal distance between sabnasal and pronasal/upper lip height; Cm-Sn-Ls = nasolabial angle; a2 = angle between horizontal plane and tangential line to labrale superius; Li-B-Pog = labiomental angle; Sn-Ls.B-Li = interlabial angle (upper and lower lip protrusion); Po-N-Pog = mandibular protrusion (angle between pogonion and porion-nasion line); B-Pog.FH.p = chin protrusion (angle between B-pogonion and line parallel to the FH line); A-N-B = soft-tissue ANB; G-Sn-Pog = facial convexity angle; N-Pog-FH = angle between the facial plane and Frankfort plane

Variables	N	ICC	P-value^*^	P-value^†^
Pn-N-Pog	24	0.956	<0.001	0.743
a1	24	0.916	<0.001	0.855
Pn-N/N-Sn	24	0.986	<0.001	0.955
Sn-Pn/Sn.St	24	0.863	<0.001	0.993
Cm-Sn-Ls	24	0.812	<0.001	0.817
a2	24	0.976	<0.001	0.976
Li-B-Pog	24	0.993	<0.001	0.913
Sn-Ls.SL-Li	24	0.837	<0.001	0.914
Po-N-Pog	24	0.954	<0.001	0.865
B-Pog.Go-Me	24	0.943	<0.001	0.846
A-N-B	24	0.834	<0.001	0.933
G-Sn-Pog	24	0.925	<0.001	0.937
N-Pog-FH	24	0.972	<0.001	0.847

Basic sample characteristics

A total of 48 patients were distributed equally into two groups according to gender. In total, 24 males and 24 females were assigned to the most and least attractive groups (12 in each group). The most and least attractive males’ mean ages were 21.5 ± 0.42 and 20.33 ± 1.07 years, respectively. The most and least attractive females’ mean age groups were 19.76 ± 0.25 and 20.75 ± 0.58 years, respectively.

The main findings of the variable measurements

There was no significant difference in frontal ratios between the most and least attractive groups regarding facial height, facial width ratios, nose width, eyes, and mouth ratios. In addition, the angles on frontal photographs showed no significant difference between the most and least attractive groups (Table 5).

**Table 5:** Descriptive statistics of the measurements of the frontal variables with the p-values of significance testing between the two groups. Bonferroni correction p-value significant at *p < 0.001. SD = standard deviation; MA = most attractive; LA = least attractive; Tr-N/N-Me = forehead height/middle and lower facial height; N-Sn/Tr-Me = nasal height/total facial height; N-Sn/N-Me = nasal height/middle and lower facial height; AlR-AlL/N-Sn = nasal width/nasal height; AlR-AlL/ChR-ChL = nasal width/mouth width; AlR-AlL/XR-XL = nasal width/facial width; EnR-EnL/XR-XL = inter-endocanthal width/facial width; EnR-EnL/ExR-ExL = inter-endocanthal width/inter-exocanthal width; EnR-EnL/AlR-AlL = inter-endocanthal width/nasal width; ChR-ChL/ExR-ExL = mouth width/inter-exocanthal width; Ls-St/St-Li = upper vermilion height/lower vermilion height; Ls-St/Sn-St = upper vermilion height/upper lip height; St-Li/St-SL = lower vermilion height/lower lip height; St-SL/Sn-St = lower lip height/upper lip height; Sn-St/ChR-ChL = upper lip height/mouth width; Cph-Li/ChR-ChL = upper and lower vermilion height/mouth width; Sn-St/Sn-Me = upper lip height/lower facial height; Sn-Me/N-Me = lower facial height/middle and lower facial height; Sn-Me/Tr-Me = lower facial height/total facial height; ChR-ChL/Sn-Me = mouth width/lower facial height; XR-XL/Tr-Me = facial width/total facial height; N-Me/XR-XL = middle and lower facial height/facial width; Sn-Me/XR-XL = lower facial height/facial width; ChR-ChL/XR-XL = mouth width/facial width; ZyR-Me-ZyL = upper facial width angle; GoR.Me.GoL = mandibular width angle; ExR-Me-ExL = facial aperture modified angle; N.F-Sn.Me = facial symmetry angle (angle between facial midline and subnasal-menton line)

Variables	MA males		LA males		P-value	MA females		LA females		P-value
	Mean	SD	Mean	SD		Mean	SD	Mean	SD	
Tr-N/N-Me	50.68	4.14	51.96	5.07	0.392	52.75	9.12	52.12	4.14	0.227
N-Sn/Tr-Me	32.43	1.4	29.64	0.75	0.051	29.93	1.19	29.32	1.9	0.141
N-Sn/N-Me	48.86	2.22	45.03	1.78	0.665	45.64	0.98	44.60	3.15	0.064
AlR-AlL/N-Sn	65.45	6.67	71.006	8.71	0.293	69.98	4.62	69.08	6.45	0.191
AlR-AlL/ChR-ChL	80.34	4.48	74.30	4.9	0.559	74.78	4.16	71.94	5.82	0.359
AlR-AlL/XR-XL	29.61	3.56	31.87	3.56	0.999	29.87	2.22	29.09	1.45	0.511
EnR-EnL/XR-XL	26.60	1.47	28.018	2.04	0.439	26.69	1.73	24.68	3.6	0.248
EnR-EnL/ExR-ExL	36.20	1.74	38.55	3.15	0.378	36.42	0.52	36.76	4.37	0.162
EnR-EnL/AlR-AlL	88.10	7.41	89.26	15.75	0.013	89.39	0.89	84.61	9.81	0.037
ChR-ChL/ExR-ExL	51.35	2.23	58.91	4.33	0.229	54.59	3.15	60.64	4.19	0.394
Ls-St/St-Li	56.11	11.82	50.87	3.74	0.053	62.69	13.51	59.01	8.3	0.244
Ls-St/Sn-St	26.22	3.7	22.80	5.65	0.324	30.16	4.12	26.86	4.61	0.981
St-Li/St-SL	60.71	10.59	58.54	14.62	0.288	62.32	3.63	60.75	5.52	0.435
St-SL/Sn-St	79.95	14.6	77.69	9.65	0.337	78.25	5.71	75.46	10.75	0.530
Sn-St/ChR-ChL	44.13	4.62	46.66	3.04	0.422	45.51	4.33	44.37	3.75	0.888
cph-Li/ChR-ChL	36.34	2.8	35.95	6.37	0.130	40.09	4.14	34.85	3.2	0.654
Sn-St/Sn-Me	34.32	4.28	36.48	3.71	0.777	35.83	1.53	34.20	1.51	0.779
Sn-Me/N-Me	51.12	2.22	54.95	1.78	0.665	54.09	1.02	55.38	3.15	0.071
Sn-Me/Tr-Me	33.95	2.04	36.21	2.26	0.776	35.52	2.81	36.44	2.61	0.942
ChR-ChL/Sn-Me	77.7	3.59	78.29	7.92	0.012	79.06	5.87	77.45	6.74	0.977
XR-XL/Tr-Me	67.32	9.38	65.99	2.4	0.191	70.12	2.32	69.37	2.67	0.965
N-Me/XR-XL	95.11	3.3	99.92	5.53	0.142	74.84	29.96	94.96	5.62	0.007
Sn-Me/XR-XL	48.60	2.08	54.94	4.21	0.042	50.60	2.33	52.65	4.97	0.194
ChR-ChL/XR-XL	37.75	2.24	42.83	2.99	0.605	39.95	2.19	40.68	4.14	0.107
ZyR-Me-ZyL	67.28	1.8	63.80	2.59	0.553	67.83	0.29	65.40	3.36	0.094
GoR.Me.GoL	103.78	2.27	97.10	0.89	0.167	103.16	1.04	99.60	3.29	0.044
ExR-Me-ExL	46.14	1.35	42.80	1.64	0.316	46.66	2.52	42.40	2.51	0.885
N.F-Sn.Me	0.57	1.13	1	1.37	0.284	0	1.44	0.83	0	0.002

Among the profile variables, the most attractive males had a significantly higher value of facial plan with Frankfort horizontal plane angle (N.Pog.FH) than the least attractive males (p < 0.001). The mean values of this angle were 80.5° in most attractive and 78.1° in the least attractive males (Table 6). However, there was no significant difference between the most and least attractive females regarding any of the profile variables.

**Table 6:** Descriptive statistics of the measurements of the profile variables with the p-values of significance testing between the two groups. Bonferroni correction p-value significant at *p < 0.001. SD = standard deviation; MA = most attractive; LA = least attractive; Pn-N-Pog = the angle between pronasal and pogonion; a1 = the angle between the nostril axis and horizontal plane; Pn-N/N-Sn = nasal tip prominence ratio (horizontal distance between pronasal and perpendicular from point nasion)/nasal height; Sn-Pn/Sn.St = the horizontal distance between subnasal and pronasal/upper lip height; Cm-Sn-Ls: = nasolabial angle; a2 = angle between horizontal plane and tangential line to labrale superius; Li-B-Pog = labiomental angle; Sn-Ls.B-Li = interlabial angle (upper and lower lip protrusion); Po-N-Pog = mandibular protrusion (angle between pogonion and porion-nasion line); B-Pog.FH.p = chin protrusion (angle between B-pogonion and line parallel to the FH line); A-N-B = soft-tissue ANB; G-Sn-Pog = facial convexity angle; N-Pog-FH = angle between the facial plane and Frankfort plane

Variables	MA males		LA males		P-value	MA females		LA females		P-value
	Mean	SD	Mean	SD		Mean	SD	Mean	SD	
Pn-N-Pog	30.42	4.36	34	3.05	0.069	32	2.54	31.6	2.64	0.846
a1	17.28	7.59	23.6	12.38	0.581	24.33	7.55	17.2	6.31	0.858
Pn-N/N-Sn	35.43	7.21	41	5.01	0.729	38.06	2.77	36.09	3.25	0.404
Sn-Pn/Sn.St	76.16	8.4	64.54	5.73	0.536	82.45	11.72	69.48	13.95	0.845
Cm-Sn-Ls	100.42	14.25	107.8	6.98	0.367	102.83	13.99	101.2	6.02	0.916
a2	83.14	6.95	84.2	9.96	0.108	78.5	8.2	84	9.24	0.350
Li-B-Pog	128.57	7.39	137	7.3	0.106	124.33	7.61	131.2	7.99	0.615
Sn-Ls.SL-Li	128.21	3.7	127.2	12.11	0.131	121.83	12.3	130.2	9.6	0.831
Po-N-Pog	68.35	4.19	68.8	4.89	0.595	72.5	3.63	70.6	3.51	0.931
B-Pog.Go-Me	81.71	4.19	92.6	4.29	0.088	80.66	10.83	85.4	6.18	0.084
A-N-B	7.92	0.5	10.5	1.52	0.734	9	1.07	9.9	0.82	0.472
G-Sn-Pog	164.8	2.94	158.6	5	0.780	161.66	4.79	161	2.74	0.403
N-Pog-FH	80.5	5.94	78.1	4.95	0.000*	83.33	3.17	82.2	0.89	0.623

## Discussion

The methodology employed in this project

Achieving facial balance requires professionals to analyze the soft tissues and be familiar with the characteristics affecting the attractiveness of the face. The facial measurements were determined on the photographs because of their reliability, remeasuring when needed, and maintaining the photos for a long time [39]. However, to obtain reliable measurements, the photographs must be taken in a standard and repeatable position. For this reason, the photographs were taken in the natural head position [33]. Images were converted to black and white to eliminate factors affecting facial aesthetics assessment, such as skin, eye, and hair color [40]. The evaluation was performed on photographs in three different modes, allowing laypersons to conduct a comprehensive aesthetic assessment of the face as displaying these photos together would achieve a more general image of the patient [41]. The VAS was used to evaluate the photographs because this method is considered simple, applicable, easy to understand, and gives independent assessments of the objects [42]. The panel of raters consisted of laypersons who had no connection with the patients and were not acquainted with orthodontics or aesthetics. This panel was selected because laypersons’ opinions are considered the most objective and unbiased [43]. In addition, Kiekens et al. indicated that seven laypersons were sufficient to gain a reliable aesthetic evaluation of photographs [44]. In this study, each photograph was evaluated 240 times to increase the precision of the results. Additionally, due to gender and racial variations, using absolute values is not recommended in the facial analysis [36]; thus, facial measurements were evaluated using angles and ratios with acceptable redefinition [18,29,34].

Frontal variables

There were no significant differences among the frontal variables between the most and least attractive groups in each gender. These results are in accordance with those of Kiekens et al., who did not find a correlation between any previous variables and facial aesthetics [29]. Further, Penna et al. compared the attractiveness of oral and chin region photographs between the most and least attractive groups in both genders and did not find a significant difference in the ratio of the mouth width to the lower third of the face and pointed out that a larger mouth does not necessarily increase the person’s facial aesthetics [45]. The findings of Malkoc and Fidanciogl showed no correlation between facial attractiveness scores and the inter-endocanthal width/nasal width ratio [46]. Moreover, studies by Jang et al. and Morosini et al. did not find a significant difference in the middle and lower facial height/width ratio between the most attractive and the least attractive female groups [47,48]. Hajeer et al. assessed facial asymmetry before and after orthognathic surgery using three-dimensional facial images. After six months of follow-up, they found no significant changes in facial asymmetry scores [49]. Wroblewska et al. reviewed studies regarding factors that may affect facial symmetry and stated that facial attractiveness is not significantly affected by asymmetry when it is within the population average, which is in line with our results [50]. Furthermore, comparing facial symmetry angles in a study by Morosini et al. between the most attractive and the least attractive females, no significant differences were found [48].

However, contrary to our results, Malkoc and Fidanciogl found a negative correlation between the ratios of lower facial height/facial width, facial height/facial width, and mouth width/lower facial height with facial aesthetics, which could be attributed to the fact that in their study, the variables were compared to their ideal values [46].

The lower face width angle increased in the most attractive group, according to the study of Morosini et al., which showed that this angle positively affected facial aesthetics [48]. Studies by Kim et al. and Jang et al. found that the linear measurement of the lower facial width (at the level of the mouth corners) was less in the Miss Korea group [47,51], contrary to our findings that did not find significant differences between the studied groups. The disagreement may be due to the cultural differences in aesthetic preferences and the method of measuring the jaw width.

Penna et al. found a significantly higher ratio of upper vermilion height/upper lip height in the attractive groups of each gender and stated that a fuller upper lip plays an important role in facial aesthetics in both genders [45]. The differences between their and our results could be that their sample consisted of perioral region photographs and did not consider the overall face view.

Profile variables

Among the profile variables, significant differences in males were found regarding the angle between the facial plane and the Frankfort plane, where it had higher values in the most attractive male group (3.07%). This finding suggests that the protrusion of the chin contributes to camouflaging mandibular retraction and adds to the masculine features in males. These results agree with the findings of Suphatheerawatr and Chamnannidiadha, which indicate that the straight profile and the slightly convex profile in males were perceived as the most attractive among the modified profiles [52]. Moreover, our results agree with Tugran et al., who found that the most attractive male profile tended to be an ideal slightly convex to straight profile [53]. However, contrary to our findings, Malkoc and Fidanciogl showed that the lower jaw position did not correlate to the aesthetics of profile in adolescents [46]. The disagreement may be due to the study method differences; in this study, the two genders were studied separately, while Malkoc and Fidanciogl studied the two genders in combination.

There were no significant differences among the profile variables between the most and least attractive females, which may be explained by the evaluation method of female profiles, which was done in a general manner without considering the profile details.

Limitations

This study included class II division 1 malocclusion patients with normal growth patterns and did not have vertical patterns due to many variables under assessment. Therefore, it is necessary to conduct additional studies on aesthetic facial measurements in different growth patterns. In addition, the results of this study are the result of the laypersons’ evaluation, and because the aesthetic standards may vary between other groups of society, the outcome of the aesthetic evaluation may change accordingly. Thus, it can be suggested to conduct a future study evaluating facial aesthetics using different panel compositions. The generalizability of the current findings is limited because the current sample was collected from one teaching hospital and was dependent on one race only. The esthetic evaluation may differ from one race to another.

## Conclusions

When analyzing the frontal and profile images, the most attractive females with class II division 1 malocclusion had similar photographic measurements to those in the least attractive group. On the contrary, the most attractive males with class II division 1 malocclusion had more protrusion in the chin than those in the least attractive group, with no significant differences in other profile and frontal variables. These findings suggest considering the chin position during the diagnosis and treatment planning of male patients with class II division 1 malocclusion.
